# Supplementary material for: Multi-parametric thrombus profiling microfluidics detects intensified biomechanical thrombogenesis associated with hypertension and aging
Source: Nat Commun. 2024 Oct 21;15:9067. doi: 10.1038/s41467-024-53069-9 (PMC11494109; doi:10.1038/s41467-024-53069-9)
Supplement: Supplementary file 3 — Reporting summary [file 41467_2024_53069_MOESM3_ESM.pdf]

nature portfolio

Corresponding author(s): Yunfeng Chen

Last updated by author(s): Jul 22, 2024

Reporting Summary

Nature Portfolio wishes to improve the reproducibility of the work that we publish. This form provides structure for consistency and transparency in reporting. For further information on Nature Portfolio policies, see our Editorial Policies and the Editorial Policy Checklist.

Statistics

For all statistical analyses, confirm that the following items are present in the figure legend, table legend, main text, or Methods section.

n/a	Confirmed	
☐	☒	The exact sample size (*n*) for each experimental group/condition, given as a discrete number and unit of measurement
☐	☒	A statement on whether measurements were taken from distinct samples or whether the same sample was measured repeatedly
☐	☒	The statistical test(s) used AND whether they are one- or two-sided<br>*Only common tests should be described solely by name; describe more complex techniques in the Methods section.*
☒	☐	A description of all covariates tested
☒	☐	A description of any assumptions or corrections, such as tests of normality and adjustment for multiple comparisons
☐	☒	A full description of the statistical parameters including central tendency (e.g. means) or other basic estimates (e.g. regression coefficient) AND variation (e.g. standard deviation) or associated estimates of uncertainty (e.g. confidence intervals)
☐	☒	For null hypothesis testing, the test statistic (e.g. *F*, *t*, *r*) with confidence intervals, effect sizes, degrees of freedom and *P* value noted<br>*Give P values as exact values whenever suitable.*
☒	☐	For Bayesian analysis, information on the choice of priors and Markov chain Monte Carlo settings
☒	☐	For hierarchical and complex designs, identification of the appropriate level for tests and full reporting of outcomes
☒	☐	Estimates of effect sizes (e.g. Cohen's *d*, Pearson's *r*), indicating how they were calculated

Our web collection on statistics for biologists contains articles on many of the points above.

Software and code

Policy information about availability of computer code

Data collection	LAS X microcope software was used to acquire all data of the stenosis assay. NI Labview 2009 (National Instrument) was used to collect the BFP results. Olympus cellSens was used to acquire BFP fluorescence images and conventional flow chamber images.
Data analysis	GraphPad Prism 10 was used for data plotting and statistical analysis. All microfluidic experiments were quantitatively analyzed using ImageJ 1.53 (Fiji, National Institutes of Health). NI Labview 2009 was used to analyze the BFP results. Matlab R2020b was used to analyze fluorescence images from fBFP experiments.

For manuscripts utilizing custom algorithms or software that are central to the research but not yet described in published literature, software must be made available to editors and reviewers. We strongly encourage code deposition in a community repository (e.g. GitHub). See the Nature Portfolio guidelines for submitting code & software for further information.

Data

Policy information about availability of data

All manuscripts must include a data availability statement. This statement should provide the following information, where applicable:

- Accession codes, unique identifiers, or web links for publicly available datasets
- A description of any restrictions on data availability
- For clinical datasets or third party data, please ensure that the statement adheres to our policy

All data supporting the findings of this study are available within the article and its supplementary files. Source data of fluid dynamics simulation generated in this

April 2023

study have been deposited in the Harvard Dataverse repository. Source data of human blood sample experiments are protected and unavailable for public deposition or upon request in accordance with the signed consent of study subjects.

Research involving human participants, their data, or biological material

Policy information about studies with human participants or human data. See also policy information about sex, gender (identity/presentation), and sexual orientation and race, ethnicity and racism.

Reporting on sex and gender	This study recruited subjects of both genders.
Reporting on race, ethnicity, or other socially relevant groupings	This study recruited subjects of different ages (18-80) and varied races (Caucasian, Asian, African American) and ethnicities (Hispanic and non-Hispanic).
Population characteristics	Healthy subjects not using any medication and hypertensive patients taking only prescribed hypertension medications (e.g., prazosin, amlodipine, enalapril) were chosen for the study. Subjects with trypanophobia history or anemia, or who have not donated blood in the past two weeks, were excluded.
Recruitment	After the notice was sent out, the first suitable volunteer that responded was always chosen as the blood donor. No bias of selection can be envisioned. All participants were provided with a verbal explanation of the study. A verbal agreement and a signed consent form were respectively collected from the participants, stating that they agree for blood donation, and for the publication of the data collected using their blood and their demographic information.
Ethics oversight	All experiments were performed in accordance with relevant guidelines and approved by Institutional Review Board of the University of Texas Medical Branch (protocol number: 22-0015) and the University of Sydney Human Research Ethics Committee (HREC, project 2023/582).

Note that full information on the approval of the study protocol must also be provided in the manuscript.

Field-specific reporting

Please select the one below that is the best fit for your research. If you are not sure, read the appropriate sections before making your selection.

☒ Life sciences ☐ Behavioural & social sciences ☐ Ecological, evolutionary & environmental sciences

For a reference copy of the document with all sections, see nature.com/documents/nr-reporting-summary-flat.pdf

Life sciences study design

All studies must disclose on these points even when the disclosure is negative.

Sample size	Sample size were chosen so that significant statistical information can be obtained between multiple experimental conditions. For all experiments involving human blood perfusion, we ensured that each condition was repeated with samples from ≥3 different donors recruited. For BFP adhesion frequency experiments, ≥3 cells from each sample were tested, and each cell-bead pair were contacted for 30 (or more) times to derive one adhesion frequency value. The BFP bond lifetime sample size was based on the pool data from BFP experiments; each force bin contained at least 50 data points, ensuring that the derived mean and variance were statistically robust.<br><br>No sample size calculation was performed.
Data exclusions	For BFP single-bond lifetime measurements, we excluded lifetime events that have significant force drift as an occasional result of hardware control failure, and those that contained obvious signatures of multiple bond rupture (Chen. et al, Force regulated conformational change of integrin $\alpha V\beta 3$. Matrix Biology 60-61:70-85, 2017).
Replication	The experimental findings were reproduced in multiple independent experiments on different days. The number of biological replicates for each data panel is indicated in the figure panel itself and methods.
Randomization	Subjects were grouped based on their health conditions (healthy or hypertensive) and age (below or above 50 years old). For the testing of antithrombotic agents, blood samples were collected from healthy young subjects with a first-come-first-serve manner, which obeyed the principle of randomization. Beads and cell samples in BFP were randomly selected during experiments.
Blinding	Blinding was performed for all the data analysis of the thrombus profiling assay.

Behavioural & social sciences study design

All studies must disclose on these points even when the disclosure is negative.

Study description	*Briefly describe the study type including whether data are quantitative, qualitative, or mixed-methods (e.g. qualitative cross-sectional, quantitative experimental, mixed-methods case study).*

Research sample	*State the research sample (e.g. Harvard university undergraduates, villagers in rural India) and provide relevant demographic information (e.g. age, sex) and indicate whether the sample is representative. Provide a rationale for the study sample chosen. For studies involving existing datasets, please describe the dataset and source.*
Sampling strategy	*Describe the sampling procedure (e.g. random, snowball, stratified, convenience). Describe the statistical methods that were used to predetermine sample size OR if no sample-size calculation was performed, describe how sample sizes were chosen and provide a rationale for why these sample sizes are sufficient. For qualitative data, please indicate whether data saturation was considered, and what criteria were used to decide that no further sampling was needed.*
Data collection	*Provide details about the data collection procedure, including the instruments or devices used to record the data (e.g. pen and paper, computer, eye tracker, video or audio equipment) whether anyone was present besides the participant(s) and the researcher, and whether the researcher was blind to experimental condition and/or the study hypothesis during data collection.*
Timing	*Indicate the start and stop dates of data collection. If there is a gap between collection periods, state the dates for each sample cohort.*
Data exclusions	*If no data were excluded from the analyses, state so OR if data were excluded, provide the exact number of exclusions and the rationale behind them, indicating whether exclusion criteria were pre-established.*
Non-participation	*State how many participants dropped out/declined participation and the reason(s) given OR provide response rate OR state that no participants dropped out/declined participation.*
Randomization	*If participants were not allocated into experimental groups, state so OR describe how participants were allocated to groups, and if allocation was not random, describe how covariates were controlled.*

Ecological, evolutionary & environmental sciences study design

All studies must disclose on these points even when the disclosure is negative.

Study description	*Briefly describe the study. For quantitative data include treatment factors and interactions, design structure (e.g. factorial, nested, hierarchical), nature and number of experimental units and replicates.*
Research sample	*Describe the research sample (e.g. a group of tagged Passer domesticus, all Stenocereus thurberi within Organ Pipe Cactus National Monument), and provide a rationale for the sample choice. When relevant, describe the organism taxa, source, sex, age range and any manipulations. State what population the sample is meant to represent when applicable. For studies involving existing datasets, describe the data and its source.*
Sampling strategy	*Note the sampling procedure. Describe the statistical methods that were used to predetermine sample size OR if no sample-size calculation was performed, describe how sample sizes were chosen and provide a rationale for why these sample sizes are sufficient.*
Data collection	*Describe the data collection procedure, including who recorded the data and how.*
Timing and spatial scale	*Indicate the start and stop dates of data collection, noting the frequency and periodicity of sampling and providing a rationale for these choices. If there is a gap between collection periods, state the dates for each sample cohort. Specify the spatial scale from which the data are taken*
Data exclusions	*If no data were excluded from the analyses, state so OR if data were excluded, describe the exclusions and the rationale behind them, indicating whether exclusion criteria were pre-established.*
Reproducibility	*Describe the measures taken to verify the reproducibility of experimental findings. For each experiment, note whether any attempts to repeat the experiment failed OR state that all attempts to repeat the experiment were successful.*
Randomization	*Describe how samples/organisms/participants were allocated into groups. If allocation was not random, describe how covariates were controlled. If this is not relevant to your study, explain why.*
Blinding	*Describe the extent of blinding used during data acquisition and analysis. If blinding was not possible, describe why OR explain why blinding was not relevant to your study.*

Did the study involve field work? ☐ Yes ☐ No

Field work, collection and transport

Field conditions	*Describe the study conditions for field work, providing relevant parameters (e.g. temperature, rainfall).*
Location	*State the location of the sampling or experiment, providing relevant parameters (e.g. latitude and longitude, elevation, water depth).*
Access & import/export	*Describe the efforts you have made to access habitats and to collect and import/export your samples in a responsible manner and in*

Access & import/export	compliance with local, national and international laws, noting any permits that were obtained (give the name of the issuing authority, the date of issue, and any identifying information).
Disturbance	Describe any disturbance caused by the study and how it was minimized.

Reporting for specific materials, systems and methods

We require information from authors about some types of materials, experimental systems and methods used in many studies. Here, indicate whether each material, system or method listed is relevant to your study. If you are not sure if a list item applies to your research, read the appropriate section before selecting a response.

Materials & experimental systems

n/a	Involved in the study
☐	☒ Antibodies
☒	☐ Eukaryotic cell lines
☒	☐ Palaeontology and archaeology
☒	☐ Animals and other organisms
☒	☐ Clinical data
☒	☐ Dual use research of concern
☒	☐ Plants

Methods

n/a	Involved in the study
☒	☐ ChIP-seq
☐	☒ Flow cytometry
☒	☐ MRI-based neuroimaging

Antibodies

Antibodies used

SZ22-FITC and P2-Alexa Fluor® 488 were from Beckman Coulter (Brea, CA, USA).
AK4-Alexa Fluor® 647 and PAC-1-Alexa Fluor® 647 were from BioLegend (San Diego, CA, USA).
AK2 and HIP-8-Alexa Fluor 488 were from ThermoFisher Scientific (Waltham, MA, USA).
MBC 370.2 was from Kerafast (Boston, MA, USA).
NMC4, 2.2.9, LJ-P5, 152B6, LJ-155B39, LJ-134B29 were from MERU VasImmune (San Diego, CA, USA).
7E9, 7E3 and 10E5 were gifts from Barry S. Coller (Rockefeller University).
RU5 was purchased from Creative Biolabs (Shirley, NY, USA).

Validation

SZ22 is a monoclonal antibody reacts with the α chain of CD41 (GPIIbα) on platelets.
Validation: Bassler, N. et al. A mechanistic model for paradoxical platelet activation by ligand-mimetic alphaIIb beta3 (GPIIb/IIIa) antagonists. Arteriosclerosis, thrombosis, and vascular biology 27, e9-15, doi:10.1161/01.ATV.0000255307.65939.59 (2007).

P2 is an integrin aIIbb3 complex–specific mAb.
Validation: The following paper describes the use of P2: Goschnick, M.W., Lau, L.M., Wee, J.L., Liu, Y.S., Hogarth, P.M., Robb, L.M., Hickey, M.J., Wright, M.D. and Jackson, D.E., 2006. Impaired "outside-in" integrin αIIbβ3 signaling and thrombus stability in TSSC6-deficient mice. Blood, 108(6), pp.1911-1918.

AK4 is a monoclonal antibody used to detect P-selectin on platelet surface.
Validation: 1. Manufacture website: https://www.biolegend.com/fr-lu/products/pe-anti-human-cd62p-p-selectin-antibody-595?GroupID=BLG15664
2. The following paper describes the use of AK4: Li, J.M., Podolsky, R.S., Rohrer, M.J., Cutler, B.S., Massie, M.T., Barnard, M.R. and Michelson, A.D., 1996. Adhesion of activated platelets to venous endothelial cells is mediated via GPIIb/IIIa. Journal of Surgical Research, 61(2), pp.543-548.

PAC-1 is against human integrin alpha IIb beta 3 in the activated form.
Validation: 1. Manufacture website: http://www.bdbiosciences.com/eu/reagents/research/clinical-research---ruo-gmp/purifiedantibodies/purified-mouse-anti-human-pac-1-pac-1/p/340535
2. The following paper describes the use of PAC-1 to detect activated integrin alpha IIb beta 3: Shattil SJ, Hoxie JA, Cunningham M, Brass LF. Changes in the platelet membrane glycoprotein IIb.IIIa complex during platelet activation. J Biol Chem 1985;260:11107–11114.

AAK2 binds to GPIbα at VWF binding site.
Validation: Manufacture website: https://www.thermofisher.com/antibody/product/CD42b-Antibody-clone-AK2-Monoclonal/MA5-16564

HIP8 is against human integrin alpha IIb.
Validation: http://www.bdbiosciences.com/eu/reagents/research/antibodies-buffers/immunology-reagents/anti-human-antibodies/cell-surface-antigens/fitc-mouse-anti-human-cd41a-hip8/p/555466

MBC370.2 is against human integrin alpha IIb and is specific for calf-1 domain when the integrin is extended.
Validation: Chen, Y., Ju, L.A., Zhou, F., Liao, J., Xue, L., Su, Q.P., Jin, D., Yuan, Y., Lu, H., Jackson, S.P. and Zhu, C., 2019. An integrin αIIbβ3 intermediate affinity state mediates biomechanical platelet aggregation. Nature materials, 18(7), pp.760-769.

NMC4 is an antibody against the A1 domain of VWF
Validation: The following paper describes the use of NMC4: Savage, B., Saldívar, E. and Ruggeri, Z.M., 1996. Initiation of platelet adhesion by arrest onto fibrinogen or translocation on von Willebrand factor. Cell, 84(2), pp.289-297.

2.2.9 is a monoclonal non-inhibitory anti-VWF antibody
Validation: The following paper describes the use of 2.2.9: Dent, J.A., Galbusera, M. and Ruggeri, Z.M., 1991. Heterogeneity of plasma von Willebrand factor multimers resulting from proteolysis of the constituent subunit. The Journal of clinical investigation, 88(3), pp.774-782.

LJ-P5 is a monoclonal antibody that blocks integrin αIIbβ3 binding to VWF but not Fg
Validation: The following paper describes the use of LJ-P5: De Marco, L., Girolami, A., Zimmerman, T. S. & Ruggeri, Z. M. von Willebrand factor interaction with the glycoprotein IIb/IIa complex. Its role in platelet function as demonstrated in patients with congenital afibrinogenemia. The Journal of clinical investigation 77, 1272-1277, doi:10.1172/JCI112430 (1986).

152B6 is a monoclonal antibody that blocks VWF binding to integrin αIIbβ3 but not GPIbα
Validation: The following paper describes the use of 152B6: Berliner, S., Niiya, K., Roberts, J. R., Houghten, R. A. & Ruggeri, Z. M. Generation and characterization of peptide-specific antibodies that inhibit von Willebrand factor binding to glycoprotein IIb-IIIa without interacting with other adhesive molecules. Selectivity is conferred by Pro1743 and other amino acid residues adjacent to the sequence Arg1744-Gly1745-Asp1746. The Journal of biological chemistry 263, 7500-7505 (1988).

7E9, LJ-155B39 and LJ-134B29 inhibit integrin αIIbβ3–Fg interaction by respectively blocking one of the three integrin-binding sites in Fg: γ408-411 (AGDV), Aα95-98 (RGDF) and Aα572-575 (RGDS).
Validation: The following paper describes the use of 7E9: Lengweiler, S. et al. Preparation of monoclonal antibodies to murine platelet glycoprotein IIb/IIIa (alphaIIbbeta3) and other proteins from hamster-mouse interspecies hybridomas. Biochemical and biophysical research communications 262, 167-173, doi:10.1006/bbrc.1999.1172 (1999).
The following paper describes the use of LJ-155B39 and LJ-134B29: Felding-Habermann, B., Ruggeri, Z. M. & Cheresh, D. A. Distinct biological consequences of integrin alpha v beta 3-mediated melanoma cell adhesion to fibrinogen and its plasmic fragments. The Journal of biological chemistry 267, 5070-5077 (1992).

Antibody 7E3 is a human platelet integrin beta 3 antagonist, the Fab fragment of which is c7E3.
Validation: 1. Clinical name of c7E3 is ReoPro™, which was approved for human use in the United States in Dec 1994. See the webpage below for details: https://www.rxlist.com/reopro-drug.htm
2. The following paper describes the inhibitory effect of c7E3 on platelet integrin alpha IIb beta 3: Coller, B.S. Platelet GPIIb/IIIa antagonists: the first anti-integrin receptor therapeutics. J Clin Invest. 1997;99(7):1467-71.

10E5 is a monoclonal antibody that blocks integrin αIIbβ3.
Validation: The following paper describes the use of 10E5: Chen, Y. et al. An integrin alphaIIbbeta3 intermediate affinity state mediates biomechanical platelet aggregation. Nature Materials 18, 760-769, doi:10.1038/s41563-019-0323-6 (2019).

RU5 is a monoclonal antibody that blocks VWF binding to collagen.
Validation: The following paper describes the use of RU5: Romijn, R.A. et al. Identification of the collagen-binding site of the von Willebrand factor A3-domain. J Biol Chem 276(13):9985-91, doi: 10.1074/jbc.M006548200 (2000).

Eukaryotic cell lines

Policy information about cell lines and Sex and Gender in Research

Cell line source(s): *State the source of each cell line used and the sex of all primary cell lines and cells derived from human participants or vertebrate models.*

Authentication: *Describe the authentication procedures for each cell line used OR declare that none of the cell lines used were authenticated.*

Mycoplasma contamination: *Confirm that all cell lines tested negative for mycoplasma contamination OR describe the results of the testing for mycoplasma contamination OR declare that the cell lines were not tested for mycoplasma contamination.*

Commonly misidentified lines (See ICLAC register): *Name any commonly misidentified cell lines used in the study and provide a rationale for their use.*

Palaeontology and Archaeology

Specimen provenance: *Provide provenance information for specimens and describe permits that were obtained for the work (including the name of the issuing authority, the date of issue, and any identifying information). Permits should encompass collection and, where applicable, export.*

Specimen deposition: *Indicate where the specimens have been deposited to permit free access by other researchers.*

Dating methods: *If new dates are provided, describe how they were obtained (e.g. collection, storage, sample pretreatment and measurement), where they were obtained (i.e. lab name), the calibration program and the protocol for quality assurance OR state that no new dates are provided.*

☐ Tick this box to confirm that the raw and calibrated dates are available in the paper or in Supplementary Information.

Ethics oversight: *Identify the organization(s) that approved or provided guidance on the study protocol, OR state that no ethical approval or guidance was required and explain why not.*

Note that full information on the approval of the study protocol must also be provided in the manuscript.

Animals and other research organisms

Policy information about studies involving animals; ARRIVE guidelines recommended for reporting animal research, and Sex and Gender in Research

Laboratory animals	*For laboratory animals, report species, strain and age OR state that the study did not involve laboratory animals.*
Wild animals	*Provide details on animals observed in or captured in the field; report species and age where possible. Describe how animals were caught and transported and what happened to captive animals after the study (if killed, explain why and describe method; if released, say where and when) OR state that the study did not involve wild animals.*
Reporting on sex	*Indicate if findings apply to only one sex; describe whether sex was considered in study design, methods used for assigning sex. Provide data disaggregated for sex where this information has been collected in the source data as appropriate; provide overall numbers in this Reporting Summary. Please state if this information has not been collected. Report sex-based analyses where performed, justify reasons for lack of sex-based analysis.*
Field-collected samples	*For laboratory work with field-collected samples, describe all relevant parameters such as housing, maintenance, temperature, photoperiod and end-of-experiment protocol OR state that the study did not involve samples collected from the field.*
Ethics oversight	*Identify the organization(s) that approved or provided guidance on the study protocol, OR state that no ethical approval or guidance was required and explain why not.*

Note that full information on the approval of the study protocol must also be provided in the manuscript.

Clinical data

Policy information about clinical studies

All manuscripts should comply with the ICMJE guidelines for publication of clinical research and a completed CONSORT checklist must be included with all submissions.

Clinical trial registration	*Provide the trial registration number from ClinicalTrials.gov or an equivalent agency.*
Study protocol	*Note where the full trial protocol can be accessed OR if not available, explain why.*
Data collection	*Describe the settings and locales of data collection, noting the time periods of recruitment and data collection.*
Outcomes	*Describe how you pre-defined primary and secondary outcome measures and how you assessed these measures.*

Dual use research of concern

Policy information about dual use research of concern

Hazards

Could the accidental, deliberate or reckless misuse of agents or technologies generated in the work, or the application of information presented in the manuscript, pose a threat to:

No	Yes	
☒	☐	Public health
☒	☐	National security
☒	☐	Crops and/or livestock
☒	☐	Ecosystems
☒	☐	Any other significant area

Experiments of concern

Does the work involve any of these experiments of concern:

No	Yes	
☒	☐	Demonstrate how to render a vaccine ineffective
☒	☐	Confer resistance to therapeutically useful antibiotics or antiviral agents
☒	☐	Enhance the virulence of a pathogen or render a nonpathogen virulent
☒	☐	Increase transmissibility of a pathogen
☒	☐	Alter the host range of a pathogen
☒	☐	Enable evasion of diagnostic/detection modalities
☒	☐	Enable the weaponization of a biological agent or toxin
☒	☐	Any other potentially harmful combination of experiments and agents

Plants

Seed stocks

Report on the source of all seed stocks or other plant material used. If applicable, state the seed stock centre and catalogue number. If plant specimens were collected from the field, describe the collection location, date and sampling procedures.

Novel plant genotypes

Describe the methods by which all novel plant genotypes were produced. This includes those generated by transgenic approaches, gene editing, chemical/radiation-based mutagenesis and hybridization. For transgenic lines, describe the transformation method, the number of independent lines analyzed and the generation upon which experiments were performed. For gene-edited lines, describe the editor used, the endogenous sequence targeted for editing, the targeting guide RNA sequence (if applicable) and how the editor was applied.

Authentication

Describe any authentication procedures for each seed stock used or novel genotype generated. Describe any experiments used to assess the effect of a mutation and, where applicable, how potential secondary effects (e.g. second site T-DNA insertions, mosiacism, off-target gene editing) were examined.

ChIP-seq

Data deposition

☐ Confirm that both raw and final processed data have been deposited in a public database such as GEO.

☐ Confirm that you have deposited or provided access to graph files (e.g. BED files) for the called peaks.

Data access links
May remain private before publication.

For "Initial submission" or "Revised version" documents, provide reviewer access links. For your "Final submission" document, provide a link to the deposited data.

Files in database submission

Provide a list of all files available in the database submission.

Genome browser session
(e.g. UCSC)

Provide a link to an anonymized genome browser session for "Initial submission" and "Revised version" documents only, to enable peer review. Write "no longer applicable" for "Final submission" documents.

Methodology

Replicates

Describe the experimental replicates, specifying number, type and replicate agreement.

Sequencing depth

Describe the sequencing depth for each experiment, providing the total number of reads, uniquely mapped reads, length of reads and whether they were paired- or single-end.

Antibodies

Describe the antibodies used for the ChIP-seq experiments; as applicable, provide supplier name, catalog number, clone name, and lot number.

Peak calling parameters

Specify the command line program and parameters used for read mapping and peak calling, including the ChIP, control and index files used.

Data quality

Describe the methods used to ensure data quality in full detail, including how many peaks are at FDR 5% and above 5-fold enrichment.

Software

Describe the software used to collect and analyze the ChIP-seq data. For custom code that has been deposited into a community repository, provide accession details.

Flow Cytometry

Plots

Confirm that:

☒ The axis labels state the marker and fluorochrome used (e.g. CD4-FITC).

☒ The axis scales are clearly visible. Include numbers along axes only for bottom left plot of group (a 'group' is an analysis of identical markers).

☒ All plots are contour plots with outliers or pseudocolor plots.

☒ A numerical value for number of cells or percentage (with statistics) is provided.

Methodology

Sample preparation	Platelet suspension was incubated with 2 μg mL-1 of HIP-8-Alexa Fluor® 488, MBC370.2-Alexa Fluor® 555, PAC-1-Alexa Fluor® 647 or AK4-Alexa Fluor® 647 for 10 min, diluted with Hepes-Tyrode buffer by 10 times, and immediately analyzed by flow cytometry.
Instrument	BD FACSymphony A5 SE
Software	BD FACSDiva; FlowJo v10 (FlowJo LLC; Ashland, OR, USA).
Cell population abundance	Sorting was not performed.
Gating strategy	Gating was not performed.

☐ Tick this box to confirm that a figure exemplifying the gating strategy is provided in the Supplementary Information.

Magnetic resonance imaging

Experimental design

Design type	*Indicate task or resting state; event-related or block design.*
Design specifications	*Specify the number of blocks, trials or experimental units per session and/or subject, and specify the length of each trial or block (if trials are blocked) and interval between trials.*
Behavioral performance measures	*State number and/or type of variables recorded (e.g. correct button press, response time) and what statistics were used to establish that the subjects were performing the task as expected (e.g. mean, range, and/or standard deviation across subjects).*

Acquisition

Imaging type(s)	*Specify: functional, structural, diffusion, perfusion.*
Field strength	*Specify in Tesla*
Sequence & imaging parameters	*Specify the pulse sequence type (gradient echo, spin echo, etc.), imaging type (EPI, spiral, etc.), field of view, matrix size, slice thickness, orientation and TE/TR/flip angle.*
Area of acquisition	*State whether a whole brain scan was used OR define the area of acquisition, describing how the region was determined.*

Diffusion MRI ☐ Used ☐ Not used

Preprocessing

Preprocessing software	*Provide detail on software version and revision number and on specific parameters (model/functions, brain extraction, segmentation, smoothing kernel size, etc.).*
Normalization	*If data were normalized/standardized, describe the approach(es): specify linear or non-linear and define image types used for transformation OR indicate that data were not normalized and explain rationale for lack of normalization.*
Normalization template	*Describe the template used for normalization/transformation, specifying subject space or group standardized space (e.g. original Talairach, MNI305, ICBM152) OR indicate that the data were not normalized.*
Noise and artifact removal	*Describe your procedure(s) for artifact and structured noise removal, specifying motion parameters, tissue signals and physiological signals (heart rate, respiration).*

Volume censoring | *Define your software and/or method and criteria for volume censoring, and state the extent of such censoring.*

Statistical modeling & inference

Model type and settings | *Specify type (mass univariate, multivariate, RSA, predictive, etc.) and describe essential details of the model at the first and second levels (e.g. fixed, random or mixed effects; drift or auto-correlation).*

Effect(s) tested | *Define precise effect in terms of the task or stimulus conditions instead of psychological concepts and indicate whether ANOVA or factorial designs were used.*

Specify type of analysis: ☐ Whole brain ☐ ROI-based ☐ Both

Statistic type for inference | *Specify voxel-wise or cluster-wise and report all relevant parameters for cluster-wise methods.*

(See Eklund et al. 2016)

Correction | *Describe the type of correction and how it is obtained for multiple comparisons (e.g. FWE, FDR, permutation or Monte Carlo).*

Models & analysis

n/a	Involved in the study
☐	☐ Functional and/or effective connectivity
☐	☐ Graph analysis
☐	☐ Multivariate modeling or predictive analysis

Functional and/or effective connectivity | *Report the measures of dependence used and the model details (e.g. Pearson correlation, partial correlation, mutual information).*

Graph analysis | *Report the dependent variable and connectivity measure, specifying weighted graph or binarized graph, subject- or group-level, and the global and/or node summaries used (e.g. clustering coefficient, efficiency, etc.).*

Multivariate modeling and predictive analysis | *Specify independent variables, features extraction and dimension reduction, model, training and evaluation metrics.*
